# Melatonin Mitigates Salt Stress in Wheat Seedlings by Modulating Polyamine Metabolism

**DOI:** 10.3389/fpls.2018.00914

**Published:** 2018-07-03

**Authors:** Qingbo Ke, Jun Ye, Bomei Wang, Jianhong Ren, Lina Yin, Xiping Deng, Shiwen Wang

**Affiliations:** ^1^State Key Laboratory of Soil Erosion and Dryland Farming on the Loss Plateau, Institute of Soil and Water Conservation, Northwest A&F University, Yangling, China; ^2^State Key Laboratory of Soil Erosion and Dryland Farming on the Loss Plateau, Institute of Soil and Water Conservation, Chinese Academy of Sciences and Ministry of Water Resources, Yangling, China; ^3^Inner Mongolia Academy of Agricultural & Animal Husbandry Sciences, Hohhot, China; ^4^College of Natural Resources and Environment, Northwest A&F University, Yangling, China; ^5^College of Life Science, Northwest A&F University, Yangling, China

**Keywords:** melatonin, wheat, polyamine, salt stress, *TaSNAT*

## Abstract

Melatonin, a small molecular weight indoleamine molecule, is involved in various biological processes and responses to environmental cues in plants. However, its function in abiotic stress response and the underlying mechanisms is less clear. In this study, we investigated the effect of melatonin on wheat seedlings growth under salt stress condition. Exogenous melatonin pretreatment partially mitigated the salt-induced inhibition of whole-plant growth as judged from shoot dry weight, IAA content, leaf photosynthesis rate, maximum photochemistry efficiency of photosystem II, and chlorophyll. The mitigation was also observed in reduced accumulation of H_2_O_2_ in melatonin-pretreated wheat seedlings exposed to salt stress. Exogenous melatonin increased endogenous melatonin content by evaluating the levels of *TaSNAT* transcript, which encodes a key regulatory enzyme in the melatonin biosynthetic pathway. Furthermore, melatonin increased polyamine contents by accelerating the metabolic flow from the precursor amino acids arginine and methionine to polyamines; melatonin also decreased the degradation of salt-induced polyamines. Taken together, these results provide the evidence that melatonin mitigates salt stress mainly through its regulation on polyamine metabolism of wheat seedlings.

## Introduction

Environmental problems such as global warming, drought, and salinity severely limit agricultural productivity in many parts of the world (Tester and Langridge, 2010). Each year there is a deterioration of 2 million hectare (about 1%) of the world agricultural lands because of salinity, leading to reduced or no plant productivity (Ke et al., 2016). High salt levels cause ion toxicity (mainly Na^+^), hyperosmotic stress, and secondary stresses such as oxidative damage and leaf senescence (Zhu, 2003). Genetic modifications that increase the activity of transporters (SOS1 and HKT1) or increase the endogenous levels of antioxidants and osmo-protectants offer a useful strategy for crop improvement (Yang et al., 2009; Ke et al., 2016; Zhu, 2016). However, because of complexities and controversies that are coupled to genetically modified crops, these genes have few applications in actual agriculture practice (Tester and Langridge, 2010). Therefore, an alternative strategy for enhancing stress tolerance and extending leaf longevity could lead to important agricultural applications.

In this context, a ubiquitous, naturally occurring biomolecule, melatonin (*N*-acetyl-5-methoxytryptamine), merits consideration. Melatonin is a low molecular weight molecule with an indole structure, which is present in all kingdoms, from prokaryotes to eukaryotes, from animals to plants (Arnao and Hernández-Ruiz, 2014). Melatonin was initially identified as an important animal hormone involved in various biological process including antioxidant actions, reproduction, circadian rhythms, and innate immunity (Pandi-Perumal et al., 2006; Tan et al., 2010; Shi et al., 2015). Since melatonin was first detected in Japanese morning glory (*Pharbitis nil*) in 1993, there has been much progress in unraveling the role of melatonin in plants (Van Tassel et al., 2001). Various studies have suggested specific physiological actions for melatonin in plants including growth promoting activity and induction of rhizogenesis, thus acting in a similar way as auxin, indolyl-3-acetic acid (IAA) (Arnao and Hernández-Ruiz, 2014). Melatonin plays a protective role against abiotic stresses, such as heavy metal, UV radiation, salt, drought and ambient temperature, and it also plays a significant role in the leaf senescence process (Reiter et al., 2015). A primary function attributed to melatonin in plants is to act as a reactive oxygen species (ROS) scavenger, serving as the first line of defense against internal and environmental oxidative stress (Park et al., 2013; Arnao and Hernández-Ruiz, 2015). Exogenously applied or endogenously induced melatonin enhances plant resistance to environmental stresses, such as drought, salt, cold, oxidative stress, and also delays leaf senescence (Li et al., 2012; Wang et al., 2013; Arnao and Hernández-Ruiz, 2014; Zhang et al., 2014). Although the action of melatonin as a possible antioxidant and plant growth regulator is still not well understood, previous studies suggested that melatonin improves the redox state of cells, decreasing ROS and reactive nitrogen species levels, and stabilizing biological membranes, as it does in animal cells (Reiter et al., 2002; Arnao and Hernández-Ruiz, 2014; Manchester et al., 2015).

Another class of biomolecule involved in plant stress response is composed of polyamines (PAs), which are low molecular weight aliphatic cations that are ubiquitous cellular components (Hussain et al., 2011). In plants, the major PAs putrescine, spermidine (spd), and spermine (spe) have been shown to be involved in many aspects of plant growth and development (such as organogenesis, embryogenesis, flower initiation and development, leaf senescence, fruit development and ripening, abiotic and biotic plant stress responses) (Bouchereau et al., 1999; Wimalasekera et al., 2011; Gupta et al., 2013). PAs can also act as anti-senescence and anti-stress agents due to their acid neutralizing and antioxidant properties, as well as for their membrane and cell wall stabilizing abilities (Yiu et al., 2009; Gupta et al., 2013). Both exogenous application and genetically engineered biosynthesis of PAs enhance the tolerance of plants to various types of abiotic stress such as salinity, cold, drought, heavy metals, osmotic stress, high temperature, water logging and flooding (Gill and Tuteja, 2010; Shi et al., 2013; Minocha et al., 2014). Recent reports indicate that exogenous melatonin increases the content of PAs in abiotic stress-treated cucumber seedlings (Zhao et al., 2017), *Arabidopsis* (Zhou et al., 2016; Zhu et al., 2016), Bermuda grass (Shi et al., 2014), carrot suspension cells (Lei et al., 2004), and harvested peach fruits (Cao et al., 2016). These studies indicate that melatonin may exert a protective effect, possibly involving accumulation of PAs. However, little is known about the regulation of melatonin-mediated PA accumulation in plants. Unraveling the mechanism underlying melatonin biosynthesis and its correlation with PA metabolism in plants could enable the application of melatonin for crop improvement and protections.

Wheat (*Triticum aestivum* L.) is one of the most important cereal staple in the world. In 2016, the global production of wheat was 749 million tones (Shin et al., 2017). Wheat provides 20% of the daily protein requirements, and calories for 4.5 billion people worldwide. However, because most wheat cultivars are extremely sensitive to saline soil (Setter et al., 2016), increasing the salt tolerance of wheat has become a major challenge for modern agriculture. In this study, we investigated the roles of melatonin in regulating salt and leaf senescence in wheat seedlings. In addition, we evaluated the contents of PAs and IAA in melatonin and salt-treated wheat seedlings. This study provides evidence that melatonin can mitigate salt stress in wheat seedlings and points to a possible link between melatonin and PA levels.

## Materials and Methods

### Plant Materials and Growth Conditions

The wheat ecotype Xinong 9871 was used in this study. Wheat seeds were sterilized with 1% sodium hypochlorite for 10 min. After washing with distilled water 3∼5 times, seeds were placed in petri plates with filter paper for 3 days to germinate. For hydroponic culture, wheat seedlings were grown on 1/4 Hoagland solution for 7 days, and then half the seedlings were supplemented with 1 μM melatonin. Three days after the pretreatment, the plants were treated with or without 100 mM NaCl for 16 days, with the media refreshed twice per week. This protocol resulted in four experimental groups of plants: (i) Control; (ii) Melatonin treatment; (iii) Salt stress treatment; (iv) Salt and melatonin treatment. All the experiments were conducted in a growth chamber at 28/23°C (day/night) with 50 ± 5% relative humidity under a light intensity of 450 μmol m^-2^ s^-1^, and a 12/12 h (light/dark) photoperiod. There were at least three biological replicates per treatment.

### Extraction and Measurement of Free IAA Contents

Extraction and quantification of endogenous IAA in plant leaves were performed according to the method described by Pan et al. (2008) and Ke et al. (2015). Briefly, ground leaves were incubated in extraction solvent (2-propanol/H_2_O/concentrated HCl, 2:1:0.002, v/v/v) with a sample:solvent ratio of 1:10 (mg/μL) on a shaker at 100 rpm for 30 min at 4°C; 1 mL dichloromethane was then added to each sample. After shaking for 30 min at 4°C, the sample was centrifuged at 13,000 *g* for 5 min at 4°C, and the lower phase was evaporated to dryness using gaseous nitrogen and re-dissolved in 0.1 mL methanol for IAA measurements. And then IAA contents were measured using an IAA ELISA kit (Shanghai Enzyme-linked Biotechnology Co., Ltd., China) according to the manufacturer’s instructions. The absorbance values at 405 nm were measured using Gen 5 Data Analysis Software (BioTek Instruments, Inc., Winooski, VT, United States). Calculation of sample IAA concentrations followed the standard cure. There were at least three biological replicates per data point.

### Measurement of Photosynthetic Rates

The photosynthetic rates were measured between 9:00 h and 11:00 h using a portable photosynthesis system (Li-6400XT, LI-COR Biosciences, Lincoln, United States). The air temperature, CO_2_ concentration and photosynthetic photon flux density in the leaf chamber were set at 28°C 450 μmol⋅mol^-1^, 1000 μmol m^-2^ s^-1^, respectively. The vapor pressure deficit was maintained at approximately 2.0 Kpa. There were at least 6 biological repeats per data point.

### Analysis of Photosynthetic Activity and Chlorophyll Contents

Photosynthetic activity in leaves was estimated based on chlorophyll fluorescence-determination of photochemical yield (Fv/Fm), which represents the maximal yield of the photochemical reaction in photosystem II (PSII), using a pulse amplitude modulated chlorophyll fluorescence meter (Imaging PAM, Walz, Effleltrich, Germany) after 30 min of dark adaption. Chlorophyll contents were measured with a portable chlorophyll meter (SPAD-502, Konica Minolta, Japan). Both of these values were detected using the fifth intact fully expanded leaves from the top of individual plants. There were at least six biological repeats per data point.

### Quantification and Detection of Hydrogen Peroxide (H_2_O_2_)

H_2_O_2_ content were measured using the protocol as described by Loreto and Velikova (2001). Leaf tissues (0.5 g) were ground well in an ice bath with 5 mL 0.1% (w/v) TCA. The extract was mixed with 0.5 mL 10 mM potassium phosphate buffer (PH 7.0) containing 1 M KI. The amount of H_2_O_2_ was determined spectrophotometrically at 390 nm by reference to a standard curve prepared with H_2_O_2_ solution. H_2_O_2_ accumulation in leaves of plants was visualized by 1 mg/mL solution of 3,3-diaminobenzidine (DAB)-HCl (PH 3.8) staining under 150 μmol m^-2^ s^-1^ light at 25°C for 6 h.

### Melatonin Extraction and Analysis

Extraction and quantification of endogenous melatonin in plant leaves were performed according to the method described by Byeon and Back (2014). Approximately 0.5 g frozen leaves were ground into powder with liquid nitrogen in a mortar, and then extracted with 5 mL chloroform at 4°C overnight. After centrifuging at 10, 000 *g* for 15 min at 4°C, the chloroform phase was evaporated to dryness using nitrogen gas. The extracts of melatonin were then dissolved in 1 mL 42% methanol and filtered through a 0.45 μm membrane filter. Aliquots of 400 μL were subjected to High Performance Liquid Chromatography (HPLC) using a fluorescence detector system (SPD-20A Prominence, Shimadzu Co., Ltd., Japan). The samples were separated on the Shim-pack VP-ODS column (3 μm, 4.6 × 150 mm, Shimadzu) with a gradient elution profile (from 42% methanol to 50% methanol in 0.1% formic acid for 27 min, then isocratic elution with 50% methanol in 0.1% formic acid for 18 min at a flow rate of 0.15 mL/min). Melatonin was detected at 280 nm excitation and 348 nm emission.

### Polyamine Analyses

Leaf samples (approximately 1 g) were ground in a mortar to a fine powder and extracted in 5 mL 5% (w/v) chilled perchloric acid (PCA). After overnight extraction at 4°C, the homogenate was centrifuged for 15 min at 15, 000 *g*. For benzoylation, 500 μL supernatant phase, containing the free polyamine fraction was mixed with 1 mL 4 N NaOH, then 10 μL benzoyl chloride was immediately added. The mixture was vortexed for 30 s and incubated at room temperature for 40 min, followed by addition of 2 mL saturated NaCl. Benzoyl-polyamines were extracted in 2 mL diethyl ether. After centrifugation at 1, 500 *g* for 15 min, 1 mL of the ether phase was collected and evaporated to dryness under nitrogen gas then re-dissolved in 1 mL methanol. After filtering through 0.45 μm membrane filter, the benzoylated samples were stored at -20°C. Polyamines were assayed by HPLC; the samples were separated on the Insertsil ODS-3 (5 μm, 4.6 × 250 mm, GL Science Inc., United States) under the program: 0 ∼ 15 min, 60% methanol; 15 ∼ 35 min, 60 ∼ 90%; 35 ∼ 45 min, 90 ∼ 60%; 45 ∼ 60 min, 60% at a flow rate of 0.8 mL min^-1^ at 35°C. Polyamine peaks were detected with a UV detector at 254 nm.

### Measurements of Arginine and Methionine

Arginine and methionine were measured by HPLC analysis after derivatization of compounds with o-phthalaldehyde (OPA) according to the method descripted by Noctor and Foyer (1998). Approximately 0.2 g frozen leaves were ground into powder with liquid nitrogen in a mortar, and then extracted with 5 mL 0.1 M HCl. On thawing, the slurry was centrifuged for 15 min and the supernatant recentrifuged for 30 min. The extracted solution was derivatized by OPA and analyzed by HPLC with fluorescence detector (RF-20AXS, Shimadzu, Japan). Separations were performed on ODS Spheri 5 column (5 μm, 4.6 × 250 mm, GL Science Inc., United States) equipped with a 15 × 3.2 mm guard column (Shim-Pack, Kyoto, Japan).

### Assays of Polyamine (PAO) and Diamine Oxidase (DAO) Activities

PAO and DAO activities in plant leaves were performed according to the method of Su et al. (2006). Briefly, leaf tissues were ground well in an ice bath with 0.1 mM potassium phosphate buffer (PH 6.5). The extract was centrifuged at 10,000 *g* for 20 min at 4°C, and 0.2 mL of the supernatant was mixed with 2.5 mL of potassium phosphate buffer (100 mM, PH 6.5), 0.2 mL 4-aminoantipyrine/N,N-dimethylaniline reaction solutions, and 0.1 mL horseradish peroxidase (250 U mL^-1^). The activity of PAO was determined by the addition of 15 μL of 20 mM putrescine as a substrate, and the activity of DAO was determined by the addition of 15 μL of 20 mM spermidine and spermine as the substrate. A 0.01 value of the changes absorbance at 555 nm was defined as one activity unit.

### RNA Preparation and Gene Expression Analysis

Total RNA was extracted from leaf samples using a TakaRa MiniBEST Plant RNA Extraction Kit (TakaRa, Dalian, China) according to the manufacturer’s protocol. For cDNA synthesis, 2 μg of total RNA was reverse transcribed using a PrimeScript^TM^ II 1st Strand cDNA Synthesis Kit (TakaRa, Dalian, China). All quantitative real-time PCR (qRT-PCR) analysis was performed with a LightCycler 480 II System (Roche, Basel, Switzerland) using a SYBR Premix Ex Taq^TM^ kit (TakaRa, Dalian, China). Gene-specific primers used in this study are *TaSNAT*-F: ACTTGGTCGCCACACTACAT, *TaSNAT*-F: TCGACAAGGACGTCCCAAAT, *TaActin3*-F: CCAGTACTGCTGACTGAGGC, *TaActin3*-R: TGTTGTGCGTCCACTAGCAT. *TaActin3* was selected as internal control according to Zhao et al. (2014). All reactions were repeated at least three times.

### Statistical Analysis

Data were statistically analyzed with Statistical Package for the Social Sciences (SPSS 19.0, SPSS Inc., Chicago, IL, United States). Means were separated using Duncan’s multiple range test at *P* = 0.05.

## Results

### Melatonin Alleviates Biomass Reduction Induced by Salt Stress in Wheat Seedlings

**FIGURE 1:**
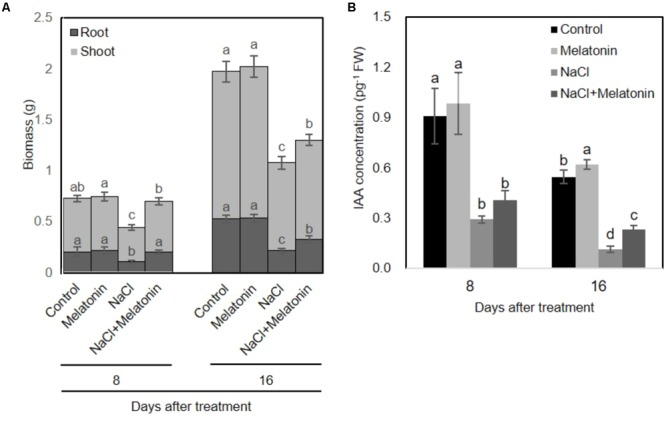
Effects of melatonin on the growth of wheat seedlings grown under control and salt stress (100 mM NaCl) conditions for 16 days. **(A)** Biomass of wheat seedlings pretreated with and without melatonin (1 μM) under salt stress (100 mM NaCl). **(B)** IAA concentrations of wheat seedlings pretreated with and without melatonin under salt stress. Data represent the mean ± SE of three biological repeats, and different lowercase letters above the bars indicating significant differences according to Duncan’s multiple range tests (*P* < 0.05), the upper error bar refers to shoot variation.

To investigate whether melatonin mitigated salt stress in wheat, we transferred the wheat seedlings to Hoagland solution containing 0 or 100 mM NaCl after pretreatment with 1 μM melatonin for 3 days. As shown in **Figure 1A**, Melatonin pretreatment has no direct discernible effects on plant growth under normal conditions. When the seedlings were subjected to salt stress for 8 days, the dry weight of non-treated wheat seedlings was significantly decreased (60% of the weight of control plants), whereas melatonin-pretreated wheat seedlings weighed in at 96.0% of the weight of the melatonin control plants. After 16 days of salt stress treatment, wheat seedlings with and without melatonin pretreatment maintained 66.1 and 54.7% lower dry biomass than plants in normal conditions.

To investigate whether melatonin affects the endogenous levels of IAA, we measured the IAA content from pretreated-wheat seedlings. As shown in **Figure 1B**, under normal conditions, exogenous melatonin-pretreatment increased endogenous IAA content in wheat seedlings. However, salt stress significantly inhibited the IAA producing, whereas these adverse effects can be alleviated on wheat seedling pretreated with melatonin. These results are consistent with the growth conditions of wheat seedlings pretreated with or without melatonin under salt stress treatment.

### Melatonin Alleviates the Inhibition of Photosynthesis in Salt-Stressed Wheat Seedlings

**FIGURE 2:**
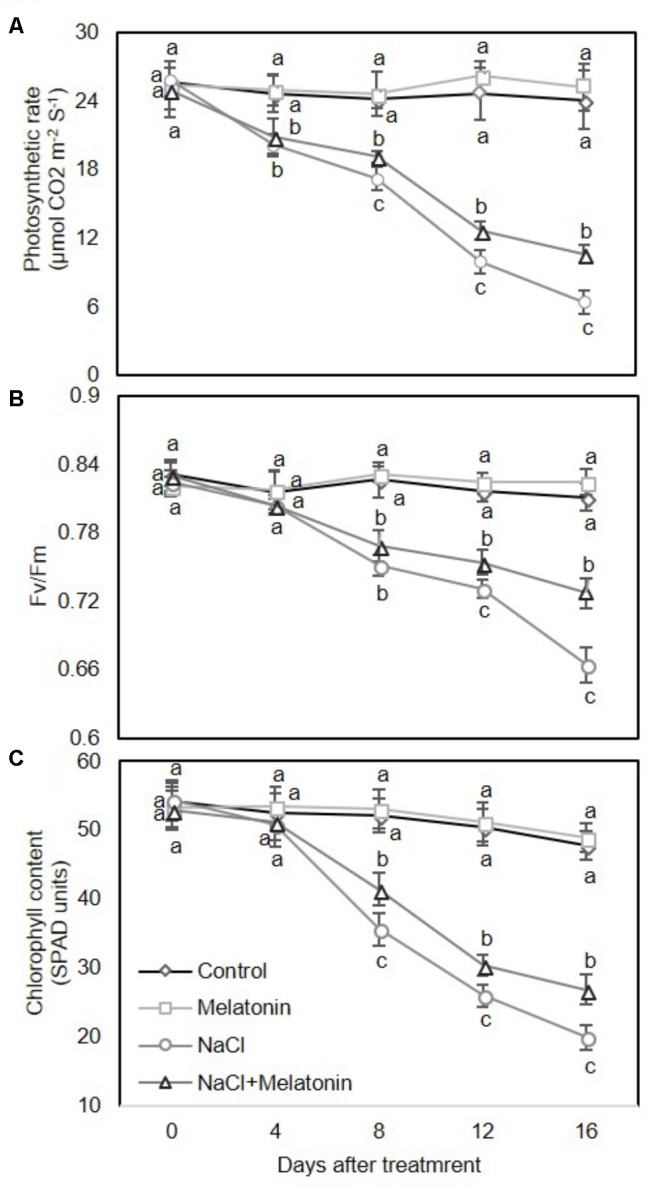
Effects of melatonin on the photosynthetic rate **(A)**, maximum efficiency of PSII photochemistry (Fv/Fm) **(B)**, and chlorophyll content **(C)** in 3rd fully expanded leaves from wheat seedlings grown under control and salt stress (100 mM NaCl) conditions for 16 days. The bars (means ± SE, *n* = 3) labeled with different letters are significantly different at *P* < 0.05 according to Duncan’s multiple range tests.

We next examined the effects of melatonin on photosynthesis and chlorophyll content in wheat seedlings under salt stress. As expected, there were no obvious upward or downward trends in leaf photosynthesis rate (Pn) (**Figure 2A**), maximum photochemistry efficiency of photosystem II (Fv/Fm) (**Figure 2B**) and chlorophyll content (**Figure 2C**) between control and melatonin pretreatment under normal conditions. Under salt stress treatment, the leaf Pn was rapidly and consistently reduced (**Figure 2A**), while the Fv/Fm and chlorophyll content in leaves decreased sharply after day 4 (**Figures 2B,C**). However, pretreatment with melatonin significantly alleviated the salt stress-induced reductions in leaf Pn, Fv/Fm and chlorophyll content. After 16 days of salt stress, Pn, Fv/Fm and chlorophyll content in wheat seedlings pretreated with melatonin were reduced by 26.6, 81.9, and 41.7%, respectively, in contrast to the in the salt stress-treated plants, accounting for 44.1, 89.6, and 56.3%, respectively (**Figure 2**). Taken together, these results suggest that exogenous melatonin treatment delays enhance salt stress tolerance of wheat seedlings.

### Melatonin Relives Salt-Induced Stress by Reducing H_2_O_2_ Produced in Wheat Seedlings

**FIGURE 3:**
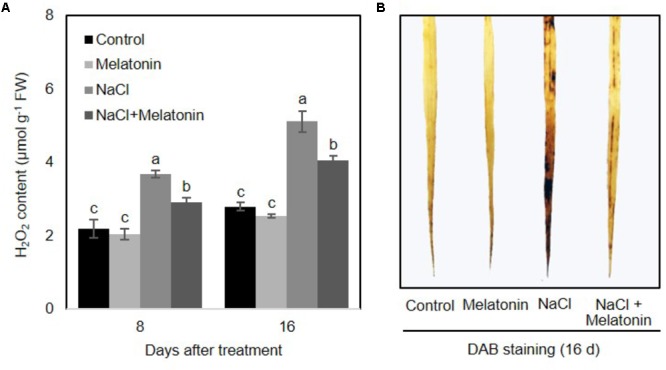
Effects of melatonin on the H_2_O_2_ accumulation in 3rd fully expanded leaves from wheat seedlings grown under control and salt stress (100 mM NaCl) conditions for 16 days. **(A)** H_2_O_2_ content in wheat seedlings leaves after salt stress, **(B)** DAB staining. Brown color indicates accumulation of H_2_O_2_. Data represent the mean ± SE of three replicate samples. Different letters indicate significant differences according to Duncan’s multiple range tests (*P* < 0.05).

To determine whether there is a link between melatonin and H_2_O_2_ scavenging, the change of H_2_O_2_ contents in wheat seedlings treated with salt stress was quantified. As shown in **Figure 3A**, under normal conditions, melatonin had no significant effects on H_2_O_2_. When salt stress was applied, H_2_O_2_ content increased consistently and significantly, although melatonin-pretreated wheat seedlings showed significantly lower levels of H_2_O_2_ (2.9 and 4.0 μmol g^-1^ after 8 and 16 days of salt stress treatment, respectively) than those of non-treated seedlings (3.6 and 5.1 μmol g^-1^ after 8 and 16 days of salt stress treatment, respectively) (**Figure 3A**). H_2_O_2_ accumulation in leaves detached from plants experiencing the four treatments was visualized by DAB staining. As expected, the detached leaves of melatonin-pretreated wheat seedlings displayed less H_2_O_2_ accumulation than those of non-treated seedlings (**Figure 3B**). These results imply that melatonin alleviates salt-induced stress in wheat seedlings by decreasing the accumulation of H_2_O_2_.

### Exogenous Melatonin Increases Endogenous Melatonin Content in Wheat Seedlings

**FIGURE 4:**
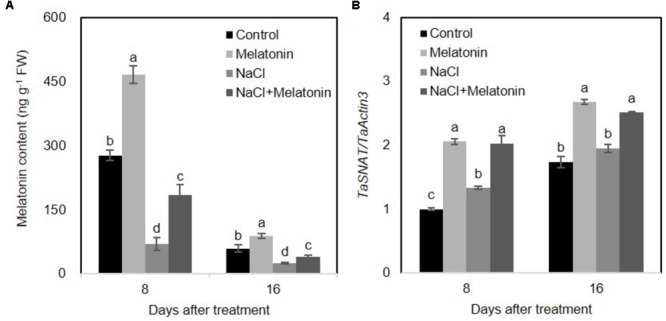
Effects of exogenous melatonin on the endogenous melatonin in wheat seedlings growth under control and salt stress (100 mM NaCl) conditions for 16 days. **(A)** Changes of endogenous melatonin in response to exogenous melatonin treatment, **(B)** expression levels of *TaSNAT* in leaves of wheat seedlings under control, exogenous melatonin (1 μM) and salt stress (100 mM NaCl) conditions for 16 days. Wheat actin 3 gene was used as an internal control. Error bars represent SE of three independent experiments. Different letters indicate significant differences at *P* < 0.05 in comparison with control.

Salt stress results in toxicity and osmotic stress in plants. Ion toxicity and osmotic stress cause metabolic imbalance, which in turn leads to oxidative stress (Chinnusamy et al., 2005). To examine the effects of exogenous melatonin pretreatment on melatonin biosynthesis, endogenous melatonin of wheat seedlings was determined using High Performance Liquid Chromatography (HPLC). As shown in **Figure 4A**, under normal conditions, exogenous melatonin pretreatment significantly increased endogenous melatonin content in wheat seedling leaves. However, the endogenous melatonin concentration markedly decreased between 8 and 16 days. Furthermore, NaCl stress prominently inhibited melatonin biosynthesis (25.2 and 42.7% compared to values in the control plants at 8 and 16 days of treatment, respectively), whereas exogenous melatonin pretreatment alleviates the inhibitory effects of high salinity on melatonin biosynthesis (in both cases, 66% of the control at 8 and 16 days of treatment) (**Figure 4A**). Quantitative RT-PCR analyses of the *TaSNAT* transcript (encoding a key regulatory enzyme in melatonin biosynthetic pathway) further demonstrated a positively correlation between exogenous melatonin treatment and the intracellular melatonin in wheat seedlings (**Figure 4B**).

### Melatonin Participates in Polyamines Metabolism

**FIGURE 5:**
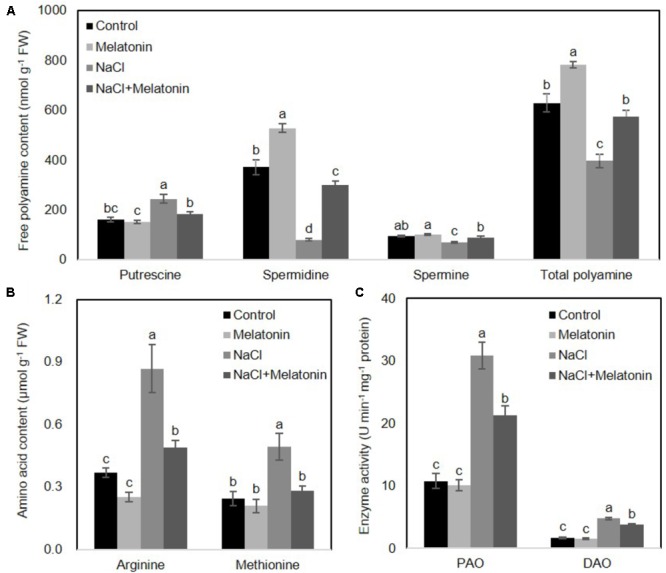
Effects of exogenous melatonin on polyamine metabolism under salt stress. **(A)** Cellular polyamine including putrescine, spermidine (Spd) and spermine (Spe), **(B)** two polyamine precursor amino acids, arginine (Arg) and methionine (Met), and **(C)** the activity of polyamine oxidase (PAO) and diamine oxidase (DAO) were measured in wheat seedlings pretreated with or without 1 μM melatonin under control and salt stress (100 mM NaCl) conditions for 16 days. Data represent the mean ± SE of three biological repeats; different lowercase letters above the bars indicating significant differences at *P* < 0.05 according to Duncan’s multiple range tests.

To determine whether the mechanisms behind the improvement of salt stress tolerance by melatonin was associated with the metabolic pathways of polyamines, the levels of polyamines were measured after 16 days of salt stress treatment. As shown in **Figure 5A**, under normal conditions, the content of spermidine (spd) and total polyamines (PAs) increased when wheat seedlings were pretreated with melatonin, as compared with non-treated seedlings, but no significant changes were found in putrescine and spermine (spe) contents. The contents of spd, spe and PAs were significantly decreased, but the putrescine level rose dramatically under salt stress. However, pretreatment with melatonin increases spd, spe and PAs contents, but decreases putrescine content when the seedlings are exposed to salt stress.

To further clarify how melatonin affects polyamine biosynthesis, two polyamine biosynthetical precursor amino acids (arginine and methionine) were quantified after 16 days of salt stress treatment. As shown in **Figure 5B**, in the non-treated wheat seedlings under normal conditions, neither arginine nor methionine contents were affected by melatonin pretreatment. However, NaCl stress resulted in increases in both arginine and methionine content, although melatonin-pretreatment resulted in significantly lower levels of arginine and methionine.

We next examined whether these alterations of polyamines were caused by activation/suppression of enzymes involved in polyamine biosynthesis. We measured the activities of polyamine oxidase (PAO) and diamine oxidase (DAO) in wheat seedlings following salt stress. As shown in **Figure 5C**, under normal conditions, melatonin has no significant effects on PAO and DAO activity. Salt stress increased PAO and DAO activity, but melatonin pretreatment prominently depressed the PAO and DAO activity. Taken together, these results suggest the protective effects of melatonin may be reflected in the polyamine levels.

## Discussion

Wheat is the most important cereal crop in the world. However, wheat productivity is restricted by multiple environmental stresses such as salinity, drought and cold (Nevo and Chen, 2010; Ke et al., 2017). We previously reported that exogenous application of melatonin increased drought stress tolerance in wheat seedlings (Ye et al., 2016). In the current study, we found that exogenous melatonin can also improve the salinity resistance of wheat seedlings. Elucidating the mechanism of how melatonin regulates plant growth and development in alleviating various abiotic stress in plants would greatly accelerate its application in crop improvement and protection.

### Melatonin Mitigates Salinity-Induced Stress in Wheat Seedlings

Melatonin as a biostimulator plays significant roles in anti-senescence and anti-stress (Arnao and Hernández-Ruiz, 2014). In this study, exogenous melatonin treatment alleviates the salinity-induced growth inhibition of wheat seedlings (**Figure 1A**). Meanwhile, exogeneous melatonin alleviates the inhibition of IAA producing in salt-stressed wheat seedlings (**Figure 1B**), which is consistent with the findings that melatonin has a stimulatory effect on root growth of mustard (*Brassica juncea*) roots, probably through melatonin-stimulated IAA biosynthesis (Chen et al., 2009). Various types of stress, such as salinity, drought, and oxidative stress severely repress the activity of photosystem II (PSII), which is determined by the balance between the rate of light-dependent repair of PSII and photoinduced damage to the PSII complex (Aro et al., 1993; Gombos et al., 1994). Melatonin seems to alleviate abiotic stress-induced inhibition partially by increasing the photosynthetic efficiency of plants. For instance, melatonin treatment improves the efficiency of PSII and alleviates the inhibition of photosynthesis in drought-stressed apple trees (Wang et al., 2013), and water-stressed (Zhang et al., 2013) or chilling-stressed cucumber seedlings (Zhao et al., 2016). Similar data were also obtained in a study of cold-stressed wheat seedlings (Turk et al., 2014). In the current study of wheat seedlings, melatonin increased the photosynthetic rate and maintained higher Fv/Fm values during salt stress (**Figure 2**), consistent with the observations of previous reports.

Multiple environmental stresses including salt, drought and high intensity light induce the overproduction of reactive oxygen species (ROS), such as superoxide radical anions, H_2_O_2_, and hydroxyl radicals, which are highly reactive and toxic, causing cell death and damage (Miller et al., 2010; Ke et al., 2015). ROS levels are primarily maintained by ROS scavenging antioxidant defense machinery (Das and Roychoudhury, 2014). It has been suggested that melatonin function as an antioxidant, providing protection against environmental agents by improving the redox state of cells, scavenging most ROS and reactive nitrogen species levels, and stabilizing biological membranes in plants (Tan et al., 2002; Arnao and Hernández-Ruiz, 2014). Here, salt stress-induced accumulation of H_2_O_2_ was suppressed by melatonin pretreatment in wheat seedling (**Figure 3**), which is consistent with the findings that melatonin decreases H_2_O_2_ accumulation in salt stress-treated watermelon (Li et al., 2017), cucumber (Zhao et al., 2016), and *Malus hupehensis* (Li et al., 2012) with respect to non-treated plants. Conceivably, melatonin possesses the ability to improve cellular redox homeostasis by activating entire antioxidant systems including antioxidant enzymes (such as catalase, superoxide dismutase, peroxidase, ascorbate peroxidase, glutathione reductase, monodehydroascorbate reductase, and dehydroascorbate reductase) and non-enzymatic antioxidants (such as glutathione and ascorbate) (Tomás-Zapico and Coto-Montes, 2005), as well as increasing levels of polyphenols (Sainz et al., 2003), carotenoids (Tan et al., 2010), and anthocyanin (Zhang et al., 2016) to protect plants from abiotic stress-induced oxidative stress. However, little is known about whether this stimulatory action is the results of the direct action of melatonin on existing enzymes or through signal transduction mechanisms which regulate gene expression and increase the production of these enzymes.

### Melatonin Alleviates Salinity-Induced Stress Possibly Mediated by Polyamine (PA) Metabolic Pathway

PAs play an important role in plant physiological and development processes by controlling cell division in rhizogenesis, embryogenesis, senescence, floral development, and fruit ripening. They also act as cell signaling molecules in helping plants to combat various abiotic stresses (Kakkar and Sawhney, 2002; Gill and Tuteja, 2010). Although the detailed mechanisms through which melatonin regulates the plant growth and abiotic stress tolerance remain unclear, several lines of evidence indicate an involvement of the metabolic pathways of PAs. Melatonin was first found to attenuate the changes in polyamine levels induced by kainite in rat brain (Lee et al., 2000). Subsequently, melatonin was shown can alleviate cold-induced apoptosis by increasing the content of putrescine and spermidine in carrot suspension cells (Lei et al., 2004). Furthermore, melatonin improves the tolerance of harvested peach fruits (Cao et al., 2016) and cucumber seedlings (Zhao et al., 2017) to chilling stress, which are associated with increased PA content. Moreover, exogenous melatonin reduces plant oxidative stress and improves iron deficiency tolerance by affecting PA metabolic pathways (Zhou et al., 2016). Similarly, in this study, we found that melatonin pretreatment significantly elevated the levels of spermidine and total PA in both normal and salt stress treatment conditions (**Figure 5A**). In addition, increased endogenies melatonin contributes to increased PA conversion from biosynthetically precursors (arginine and methionine) and reduced salt-induced polyamine degradation (**Figures 4, 5B,C**). Therefore, it seems reasonable to speculate that melatonin confers enhanced salt tolerance to wheat seedlings, possibly through regulation of PA metabolism.

Furthermore, we measured the relative expression levels of genes coding for arginine decarboxylase, spermidine synthase, spermine synthase and 1-aminocyclopropane-carboxylic acid synthase (a rate-limiting step in ethylene biosynthesis) in wheat seedlings pretreated with or without 1 μM melatonin under control and salt stress conditions for 8 and 16 days. However, there were no significantly patterns among them (data not shown). We speculate that melatonin plays roles as multi-functional biomolecule, in macromolecule and membrane stabilization, or even as enzyme protectants and antioxidants, are not necessarily by directly regulating the expression levels of related-genes (such as genes involved in polyamine catabolism), it may promote the enzymes activity by physically scavenging of reactive oxygen (ROS) or activating the ROS scavenging antioxidant defense machinery in response to various environmental stresses. Additionally, we noticed that the ethylene and polyamines share a common precursor, S-adenosyl-L-methionine, and the biosynthetic relationship between these molecules is most often considered in terms of a competitive demand, however, Quinet et al. (2010) reported that no direct antagonism between polyamines and ethylene pathways in rice, suggesting that there exists a complex network among melatonin, polyamines and ethylene catabolism. To clarify the crosstalk among melatonin, polyamines and ethylene, we are intending to investigate the genetic evidence using different inhibitors of PAs and ethylene, or corresponding mutants.

## Conclusion

**FIGURE 6:**
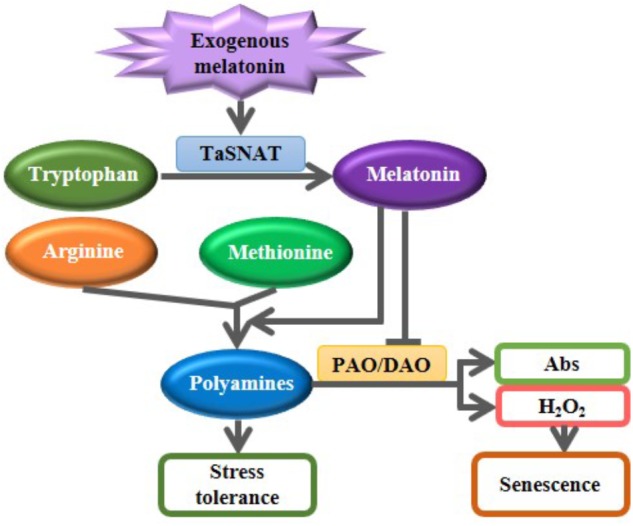
A proposed model illustrating the roles of exogenous melatonin in regulating polyamine metabolism in response to salt stress. Exogenous melatonin induces the biosynthesis of endogenous melatonin. Increased melatonin further accelerated the metabolic flow from the precursors amino acids arginine and methionine to polyamines; melatonin also decreased the degradation of salt-induced polyamines by suppressing the polyamine oxidase (PAO) and diamine oxidase (DAO) activities. Increased PAs further improve tolerance to abiotic stress in wheat seedlings. Abs, Amino butanals.

Exogenous melatonin pretreatment partially mitigated the salt-induced inhibition of whole-plant growth. Melatonin is speculated to participate in alleviating salt-induced stress in wheat seedlings, as depicted in **Figure 6**. Exogenous melatonin induces the expression of *SNAT*, which encodes the rate-limiting enzyme of the melatonin biosynthetic pathway, and subsequently increases the biosynthesis of endogenous melatonin. Melatonin further accelerated the metabolic flow from the precursors amino acids arginine and methionine to polyamines; melatonin also decreased the degradation of salt-induced polyamines by suppressing the PAO and DAO activities. Increased PAs further improve tolerance to abiotic stress in wheat seedlings. Although the proposed mechanisms of melatonin-mediated abiotic stress tolerance must be further characterized in other plants, melatonin represents a promising candidate agent for use in crop improvement and protection.

## Author Contributions

SW, QK, and JY conceived and designed the experiments. QK, JY, and BW performed the experiments. BW and JR analyzed the data. XD and LY contributed reagents, materials, and analysis tools. QK, SW, and JY wrote the paper.

## Conflict of Interest Statement

The authors declare that the research was conducted in the absence of any commercial or financial relationships that could be construed as a potential conflict of interest.
